# Supplementary material for: Development and validation of the sedentary behavior regulation scale in Korean Adults Population
Source: PLoS One. 2026 Apr 13;21(4):e0346963. doi: 10.1371/journal.pone.0346963 (PMC13075669; doi:10.1371/journal.pone.0346963)
Supplement: S1 Table — (DOCX) [file pone.0346963.s001.docx]

**Supplementary Table 1. Characteristics of the Expert Panel**

| **Expert ID** | **Discipline** | **Position** | **Years of experience** | **Affiliation type** | **Selection criteria** |
| --- | --- | --- | --- | --- | --- |
| E1 | Sports Medicine | Professor | ≥10 years | University | Physical activity & sedentary behavior research |
| E2 | Psychology | Professor | ≥10 years | University | Health behavior & self-regulation |
| E3 | Nursing | Professor | ≥10 years | University | Instrument development |
| E4 | Nursing | Nursing manager | ≥20 years | General hospital | Patient education expertise |
| E5 | Nursing | Head nurse | ≥20 years | Tertiary hospital | Clinical expertise in chronic disease |
| E6 | Nursing | Nursing education leader | ≥20 years | University hospital | Experience in curriculum and assessment |
| E7 | Medicine | Cardiologist | ≥10 years | University hospital | Cardiovascular risk factors |
