# Supplementary material for: Development and validation of the sedentary behavior regulation scale in Korean Adults Population
Source: PLoS One. 2026 Apr 13;21(4):e0346963. doi: 10.1371/journal.pone.0346963 (PMC13075669; doi:10.1371/journal.pone.0346963)
Supplement: S2 Table — (DOCX) [file pone.0346963.s002.docx]

**Supplementary Table 2. Content Validity Indices and Item Reduction Process**

| **Initial No.** | **Original**  **Component** | **Item content** | **E1** | **E2** | **E3** | **E4** | **E5** | **E6** | **E7** | **CVI** | **Decision**  **1st** | **Item**  **No** |
| --- | --- | --- | --- | --- | --- | --- | --- | --- | --- | --- | --- | --- |
| 1 | Self-awareness | I feel that prolonged sitting is harmful to my health. | 3 | 4 | 3 | 4 | 3 | 4 | 3 | 1.00 | Deleted  (duplicate) | ― |
| 2 | Self-awareness | I am concerned that my sitting habits are harmful to my health. | 3 | 4 | 3 | 3 | 4 | 4 | 4 | 1.00 | Retained | x1 |
| 3 | Self-awareness | I experience discomfort in my lower back, neck, or shoulders when sitting for long periods. | 4 | 4 | 2 | 4 | 4 | 4 | 4 | 0.86 | Retained | x2 |
| 4 | Self-awareness | I notice a decline in my concentration or vitality as my sitting time increases. | 4 | 4 | 3 | 4 | 4 | 4 | 4 | 1.00 | Modified | x3 |
| 5 | Self-awareness | I notice that my posture deteriorates the longer I remain seated. | 3 | 4 | 3 | 4 | 4 | 4 | 4 | 1.00 | Modified | x4 |
| 6 | Self-awareness | I experience stiffness or discomfort after watching TV or driving for a long time. | 3 | 2 | 1 | 4 | 3 | 4 | 3 | 0.71 | Deleted  (CVI < 0.8) | ― |
| 7 | Self-awareness | I feel a sense of heaviness in my body after sitting for a long time. | 4 | 3 | 3 | 4 | 4 | 4 | 4 | 1.00 | Retained | x5 |
| 8 | Self-awareness | I experience stiffness or a "locked" feeling in my body when standing up after prolonged sitting. | 4 | 4 | 3 | 4 | 4 | 4 | 4 | 1.00 | Retained | x6 |
| 9 | Self-awareness | I notice that I am spending more and more time sitting in my daily life. | 3 | 4 | 4 | 3 | 4 | 4 | 4 | 1.00 | Modified | x7 |
| 10 | Self-awareness | I am aware that my sitting time differs between weekdays and weekends. | 3 | 3 | 1 | 4 | 2 | 2 | 2 | 0.43 | Deleted  (CVI < 0.8) | ― |
| 11 | Behavioral practice | I strive to maintain proper posture while working. | 3 | 4 | 4 | 4 | 4 | 4 | 4 | 1.00 | Retained | x8 |
| 12 | Behavioral practice | I stand up and move when I feel discomfort in my back or neck while working. | 4 | 4 | 4 | 4 | 4 | 4 | 4 | 1.00 | Retained | x9 |
| 13 | Behavioral practice | I take breaks and stand up at regular intervals during work. | 3 | 4 | 4 | 4 | 4 | 4 | 4 | 1.00 | Retained | x10 |
| 14 | Behavioral practice | I make it a point to stand up and move at regular intervals during long periods of computer work. | 4 | 4 | 2 | 3 | 2 | 2 | 4 | 0.57 | Deleted  (CVI < 0.8) | ― |
| 15 | Behavioral practice | I bend or move my legs to promote blood circulation while sitting. | 4 | 4 | 3 | 4 | 4 | 3 | 3 | 1.00 | Retained | x11 |
| 16 | Behavioral practice | I intentionally move my legs or feet while sitting. | 3 | 4 | 2 | 3 | 2 | 3 | 3 | 0.71 | Deleted  (CVI < 0.8) | ― |
| 17 | Behavioral practice | I frequently move my legs by crossing them alternately while sitting. | 2 | 3 | 3 | 2 | 1 | 3 | 2 | 0.43 | Deleted  (CVI < 0.8) | ― |
| 18 | Behavioral practice | I lightly fidget or shift my body while in a seated position. | 2 | 3 | 4 | 3 | 4 | 3 | 4 | 0.86 | Deleted  (duplicate) | ― |
| 19 | Behavioral practice | I stand up and stretch to avoid staying the same posture for a long time. | 4 | 4 | 4 | 4 | 4 | 4 | 4 | 1.00 | Modified | x12 |
| 20 | Behavioral practice | I self-regulate my sitting time to ensure it does not exceed a certain duration. | 4 | 4 | 3 | 4 | 3 | 3 | 4 | 1.00 | Deleted  (duplicate) | ― |
| 21 | Behavioral practice | I perform simple tasks, such as organizing documents or talking on the phone, while standing. | 4 | 4 | 4 | 4 | 4 | 4 | 4 | 1.00 | Retained | x13 |
| 22 | Behavioral practice | I stand up and move after a rest stop during long drives. | 4 | 3 | 3 | 4 | 4 | 4 | 4 | 1.00 | Modified | x14 |
| 23 | Behavioral practice | I frequently stand up and move around while watching TV for long periods. | 3 | 4 | 3 | 3 | 4 | 4 | 4 | 1.00 | Modified | x15 |
| 24 | Behavioral practice | I try to maintain good posture without leaning against the sofa or wall when watching TV. | 4 | 4 | 3 | 4 | 3 | 4 | 4 | 1.00 | Retained | x16 |
| 25 | Behavioral practice | I take care not to remain seated for too long during breaks or meals. | 4 | 4 | 3 | 2 | 4 | 4 | 4 | 0.86 | Retained | x17 |
| 26 | Behavioral practice | I make an effort to stand and move around indoors, even for short durations. | 4 | 3 | 4 | 3 | 4 | 4 | 4 | 1.00 | Modified | x18 |
| 27 | Behavioral practice | I perform stretches to relieve physical tension or discomfort. | 4 | 4 | 4 | 4 | 4 | 4 | 4 | 1.00 | Retained | x19 |
| 28 | Behavioral practice | I stand up and move frequently whenever I feel physical pressure. | 2 | 3 | 4 | 4 | 4 | 4 | 4 | 0.86 | Retained | x20 |
| 29 | Environmental design | I try to improve my work environment to reduce the amount of time I spend sitting. | 3 | 3 | 2 | 4 | 3 | 4 | 4 | 0.86 | Retained | x21 |
| 30 | Environmental design | I intentionally schedule standing work time to reduce sitting time. | 2 | 4 | 3 | 4 | 4 | 4 | 4 | 0.86 | Retained | x22 |
| 31 | Environmental design | I adjust my chair or desk to reduce the strain on my lower back and neck. | 2 | 4 | 3 | 4 | 4 | 4 | 4 | 0.86 | Retained | x23 |
| 32 | Environmental design | I check my surroundings in advance to ensure a comfortable sitting environment. | 2 | 3 | 1 | 4 | 3 | 4 | 1 | 0.57 | Deleted  (CVI < 0.8) | ― |
| 33 | Environmental design | I adjust the angle of the chair's backrest before sitting to maintain proper posture. | 2 | 4 | 3 | 4 | 4 | 3 | 4 | 0.86 | Deleted  (duplicate) | ― |
| 34 | Environmental design | I set the height of my desk and monitor to suit my physical needs. | 2 | 4 | 3 | 4 | 4 | 4 | 4 | 0.86 | Retained | x24 |
| 35 | Environmental design | I adjust the placement of my laptop, mouse, and phone to reduce discomfort while sitting. | 3 | 4 | 1 | 4 | 4 | 4 | 4 | 0.86 | Removed  (lack of relevance) | ― |
| 36 | Environmental design | I adjust environmental factors, such as lighting and temperature, to minimize discomfort while sitting. | 3 | 3 | 2 | 4 | 4 | 4 | 3 | 0.86 | Removed  (lack of relevance) | ― |
| 37 | Environmental design | I prepare my environment in advance when I anticipate sitting for an extended period. | 2 | 3 | 2 | 1 | 3 | 3 | 3 | 0.57 | Deleted  (CVI < 0.8) | ― |
| 38 | Environmental design | I use a backrest or cushion to minimize pressure on my back or neck when sitting for long periods. | 2 | 4 | 4 | 4 | 4 | 3 | 4 | 0.86 | Retained | x25 |
| 39 | Environmental design | I use comfortable clothing or supportive devices (e.g., shoes, back support) when I have to sit for long periods. | 4 | 4 | 3 | 4 | 3 | 4 | 4 | 1.00 | Modified | x26 |
| 40 | Environmental design | I keep and use stretching tools or equipment (e.g., gym ball, foam roller) nearby. | 4 | 4 | 3 | 2 | 4 | 3 | 3 | 0.86 | Modified | x27 |
| 41 | Environmental design | I set alarms to remind myself to change my posture at regular intervals. | 4 | 4 | 4 | 4 | 4 | 3 | 3 | 1.00 | Retained | x28 |
| 42 | Environmental design | I vary my sitting spaces (e.g., switching between a chair, sofa, or different seating positions). | 4 | 4 | 2 | 2 | 3 | 3 | 3 | 0.71 | Deleted  (CVI < 0.8) | ― |
| 43 | Behavioral practice | I consciously plan to spend part of my day standing or moving. | 4 | 4 | 3 | 4 | 3 | 3 | 3 | 1.00 | Deleted  (duplicate) | ― |
| 44 | Behavioral practice | I try to use the stairs instead of the elevator. | 4 | 4 | 1 | 4 | 3 | 2 | 4 | 0.71 | Deleted  (CVI < 0.8) | ― |
| 45 | Behavioral practice | I choose to walk instead of drive for short distances. | 4 | 2 | 2 | 4 | 4 | 2 | 4 | 0.57 | Deleted  (CVI < 0.8) | ― |
| 46 | Behavioral practice | I try to walk or ride a bicycle when traveling short distances. | 4 | 2 | 1 | 3 | 2 | 4 | 4 | 0.57 | Deleted  (CVI < 0.8) | ― |
| 47 | Behavioral practice | I prepare activities that allow me to move intermittently when I must sit for long periods. | 4 | 4 | 2 | 3 | 4 | 4 | 4 | 0.86 | Retained | x29 |
| 48 | Behavioral practice | I try to reduce my sitting time through regular light exercise or outdoor activities. | 4 | 4 | 3 | 4 | 4 | 4 | 4 | 1.00 | Retained | x30 |
| 49 | Behavioral practice | I strive to engage in physically active outdoor activities rather than indoor activities during the weekend. | 4 | 3 | 1 | 4 | 4 | 4 | 4 | 0.86 | Removed  (lack of relevance) | ― |
| 50 | Behavioral practice | I check myself to see how much time I spend sitting during the day. | 4 | 3 | 3 | 2 | 4 | 4 | 4 | 0.86 | Modified | x31 |
| 51 | Behavioral practice | I set and implement goals to reduce the amount of time I spend sitting. | 4 | 4 | 4 | 4 | 4 | 4 | 4 | 1.00 | Retained | x32 |
