# Supplementary material for: Development and validation of the sedentary behavior regulation scale in Korean Adults Population
Source: PLoS One. 2026 Apr 13;21(4):e0346963. doi: 10.1371/journal.pone.0346963 (PMC13075669; doi:10.1371/journal.pone.0346963)
Supplement: S3 Table — (DOCX) [file pone.0346963.s003.docx]

**Supplementary Table 3.** **Items Excluded Based on Item-Total Correlation Coefficient and EFA Results**

| **Item**  **No** | **Original**  **Component** | **Item content** | **Item-total correlation coefficient** | **Communality** | **1st**  **Factor**  **loading** | **2nd**  **Factor**  **loading** | **3rd**  **Factor**  **loading** | **Decision**  **2nd** |
| --- | --- | --- | --- | --- | --- | --- | --- | --- |
| x1 | Self-  awareness | I am concerned that my sitting habits are harmful to my health. | 0.18 | ― | ― | ― | ― | Deleted  (low item–total correlation) |
| x2 | Self-  awareness | I experience discomfort in my lower back, neck, or shoulders when sitting for long periods. | 0.03 | ― | ― | ― | ― | Deleted  (low item–total correlation) |
| x3 | Self-  awareness | I notice a decline in my concentration or vitality as my sitting time increases. | 0.13 | ― | ― | ― | ― | Deleted  (low item–total correlation) |
| x4 | Self-  awareness | I notice that my posture deteriorates the longer I remain seated. | 0.07 | ― | ― | ― | ― | Deleted  (low item–total correlation) |
| x5 | Self-  awareness | I feel a sense of heaviness in my body after sitting for a long time. | 0.15 | ― | ― | ― | ― | Deleted  (low item–total correlation) |
| x6 | Self-awareness | I experience stiffness or a "locked" feeling in my body when standing up after prolonged sitting. | 0.08 | ― | ― | ― | ― | Deleted  (low item–total correlation) |
| x7 | Self-  awareness | I notice that I am spending more and more time sitting in my daily life. | 0.07 | ― | ― | ― | ― | Deleted  (low item–total correlation) |
| x8 | Behavioral practice | I strive to maintain proper posture while working. | 0.45 | 0.28 | 0.34 | - | - | Deleted  (low communality) |
| x9 | Behavioral practice | I stand up and move when I feel discomfort in my back or neck while working. | 0.48 | 0.51 | 0.81 | 0.77 | 0.77 | Retained |
| x10 | Behavioral practice | I take breaks and stand up at regular intervals during work. | 0.51 | 0.59 | 0.82 | 0.78 | 0.78 | Retained |
| x11 | Behavioral practice | I bend or move my legs to promote blood circulation while sitting. | 0.47 | 0.39 | 0.64 | 0.58 | 0.59 | Retained |
| x12 | Behavioral practice | I stand up and stretch to avoid staying the same posture for a long time. | 0.64 | 0.70 | 0.85 | 0.77 | 0.76 | Retained |
| x13 | BP | I perform simple tasks, such as organizing documents or talking on the phone, while standing. | 0.56 | 0.40 | 0.37 | 0.37 | 0.42/  0.37 | Deleted  (cross-loading on two factors) |
| x14 | Behavioral practice | I stand up and move after a rest stop during long drives. | 0.53 | 0.35 | 0.36 | 0.49 | 0.48 | Retained |
| x15 | Behavioral practice | I frequently stand up and move around while watching TV for long periods. | 0.59 | 0.42 | 0.35 | 0.47 | 0.46 | Retained |
| x16 | Behavioral practice | I try to maintain good posture without leaning against the sofa or wall when watching TV. | 0.53 | 0.47 | 0.56 | 0.53 | 0.54 | Retained |
| x17 | Behavioral practice | I take care not to remain seated for too long during breaks or meals. | 0.60 | 0.56 | 0.80 | 0.43 | 0.31 | Deleted  (low factor loading) |
| x18 | Behavioral practice | I make an effort to stand and move around indoors, even for short durations. | 0.66 | 0.66 | 0.81 | 0.58 | 0.54 | Retained |
| x19 | Behavioral practice | I perform stretches to relieve physical tension or discomfort. | 0.42 | 0.27 | 0.35 | ― | ―- | Deleted  (low communality) |
| x20 | Behavioral practice | I stand up and move frequently whenever I feel physical pressure. | 0.46 | 0.37 | 0.42 | 0.67 | 0.64 | Retained |
| x21 | Environmental design | I try to improve my work environment to reduce the amount of time I spend sitting. | 0.65 | 0.50 | 0.32 | 0.43 | 0.44/  0.35 | Deleted  (cross-loading on two factors) |
| x22 | Environmental design | I intentionally schedule standing work time to reduce sitting time. | 0.63 | 0.50 | 0.44 | 0.48 | 0.49 | Retained |
| x23 | Environmental design | I adjust my chair or desk to reduce the strain on my lower back and neck. | 0.55 | 0.67 | 0.84 | 0.36 | 0.32 | Deleted  (low factor loading) |
| x24 | Environmental design | I set the height of my desk and monitor to suit my physical needs. | 0.34 | 0.36 | 0.68 | 0.22 | ― | Deleted  (low factor loading) |
| x25 | Environmental design | I use a backrest or cushion to minimize pressure on my back or neck when sitting for long periods. | 0.51 | 0.32 | 0.36 | 0.27 | ― | Deleted  (low factor loading) |
| x26 | Environmental design | I wear comfortable clothing or supportive devices (e.g., shoes, back support) when I have to sit for long periods. | 0.54 | 0.36 | 0.50 | 0.55 | 0.54 | Retained |
| x27 | Environmental design | I keep and use stretching tools or equipment (e.g., gym ball, foam roller) nearby. | 0.56 | 0.41 | 0.47 | 0.54 | 0.54 | Retained |
| x28 | Environmental design | I set alarms to remind myself to change my posture at regular intervals. | 0.50 | 0.64 | 0.91 | 0.86 | 0.83 | Retained |
| x29 | Behavioral practice | I prepare activities that allow me to move intermittently when I must sit for long periods. | 0.62 | 0.53 | 0.46 | 0.59 | 0.59 | Retained |
| x30 | Behavioral practice | I try to reduce my sitting time through regular light exercise or outdoor activities. | 0.64 | 0.53 | 0.32 | 0.41 | 0.37/  0.43 | Deleted  (cross-loading on two factors) |
| x31 | Behavioral practice | I check myself to see how much time I spend sitting during the day. | 0.58 | 0.75 | 0.93 | 0.99 | 0.96 | Retained |
| x32 | Behavioral practice | I set and implement goals to reduce the amount of time I spend sitting. | 0.57 | 0.74 | 0.92 | 0.95 | 0.93 | Retained |

Note. Self-awareness items were removed from the final factor structure because of low item–total correlations (≤ .30).
