# Supplementary material for: Development and validation of the sedentary behavior regulation scale in Korean Adults Population
Source: PLoS One. 2026 Apr 13;21(4):e0346963. doi: 10.1371/journal.pone.0346963 (PMC13075669; doi:10.1371/journal.pone.0346963)
Supplement: S4 Table — (DOCX) [file pone.0346963.s004.docx]

**Supplementary Table 4. Final Version of the Sedentary Behavior Regulation Scale (SBRS)**

| **Factor** | **No.** | **Item** | **Strongly disagree** | **Disagree** | **Neutral** | **Agree** | **Strongly agree** |
| --- | --- | --- | --- | --- | --- | --- | --- |
| Sedentary behavior management and environmental support | 1 | I set and implement goals to reduce the amount of time I spend sitting. | ① | ② | ③ | ④ | ⑤ |
|  | 2 | I prepare activities that allow me to move intermittently when I must sit for long periods. | ① | ② | ③ | ④ | ⑤ |
|  | 3 | I keep and use stretching tools or equipment (e.g., gym ball, foam roller) nearby. | ① | ② | ③ | ④ | ⑤ |
|  | 4 | I try to maintain good posture without leaning against the sofa or wall when watching TV. | ① | ② | ③ | ④ | ⑤ |
|  | 5 | I wear comfortable clothing or supportive devices (e.g., shoes, back support) when I have to sit for long periods. | ① | ② | ③ | ④ | ⑤ |
|  | 6 | I intentionally schedule standing work time to reduce sitting time. | ① | ② | ③ | ④ | ⑤ |
| Active movement in sedentary contexts | 7 | I take breaks and stand up at regular intervals during work. | ① | ② | ③ | ④ | ⑤ |
|  | 8 | I stand up and move when I feel discomfort in my back or neck while working. | ① | ② | ③ | ④ | ⑤ |
|  | 9 | I stand up and stretch to avoid staying the same posture for a long time. | ① | ② | ③ | ④ | ⑤ |
|  | 10 | I stand up and move frequently whenever I feel physical pressure. | ① | ② | ③ | ④ | ⑤ |
|  | 11 | I bend or move my legs to promote blood circulation while sitting. | ① | ② | ③ | ④ | ⑤ |
|  | 12 | I stand up and move after a rest stop during long drives. | ① | ② | ③ | ④ | ⑤ |
