# Supplementary material for: Development and validation of the sedentary behavior regulation scale in Korean Adults Population
Source: PLoS One. 2026 Apr 13;21(4):e0346963. doi: 10.1371/journal.pone.0346963 (PMC13075669; doi:10.1371/journal.pone.0346963)
Supplement: Attachment — Submitted filename: Response to reviewer_0217.docx [file pone.0346963.s006.docx]

**Dea****r Editor and Reviewers,**

We wish to thank you for your thoughtful comments and valuable feedback on the manuscript originally titled, “**Development and Validation of the Sedentary Behavior Regulation Scale**”.

We have modified the manuscript according to your suggestions, rewriting and rephrasing sections to improve clarity, adding further information, and explaining in detail the points that were previously vague. For your convenience, we have set the revisions in the manuscript in red. We believe that the revised version of this paper will interest the readership of the PLOS ONE.

We have seriously considered the reviewers’ comments and carefully revised the manuscript. Please find our responses to the reviewer’s comments point by point below.

-----------------------------------------------------------------------------------------------------

**Reviewer 1’s comment**

**Point 1:** 1. Introduction. The introduction contextualises the construct appropriately. However, it would be advisable to update some references and further strengthen the justification for the instrument in terms of the knowledge gap and practical need.

- **Response:** We appreciate the reviewer’s thoughtful observation regarding the need to update key references and to further clarify the knowledge gap and practical necessity of the proposed instrument. In response, we revised the Introduction to incorporate up-to-date, authoritative references that currently serve as reference standards in sedentary behavior research, explicitly articulate the conceptual and methodological gap in existing sedentary behavior measures, and clarify the theoretical and practical contributions of the proposed Sedentary Behavior Regulation Scale (SBRS).
- **Line 47-54:** Sedentary behavior has become pervasive in modern society, posing a significant and independent risk factor for physical and mental health [1]. This behavior is defined as any waking behavior characterized by an energy expenditure of 1.5 metabolic equivalents (METs) or less while sitting, reclining, or lying down. A growing body of evidence recognizes sedentary behavior as an independent health risk factor, distinct from the mere lack of physical activity [2]. Recent global guidelines and meta-analytic evidence have further reinforced sedentary behavior as a standalone behavioral risk factor, irrespective of engagement in moderate-to-vigorous physical activity [3, 4].
- **Line 69-87:** Research has consistently demonstrated that increased sedentary behavior is a major risk factor for the development of various chronic diseases. Prolonged and uninterrupted sedentary time triggers a cascade of physiological changes, including insulin resistance, vascular dysfunction, altered carbohydrate metabolism, and chronic low-grade inflammation [2]. It is also associated with a shift from oxidative to glycolytic muscle fibers, reduced cardiopulmonary function, and decreased muscle and bone masses. Furthermore, it contributes to increased body and visceral fat, elevated blood lipid levels, and heightened inflammatory response [3]. Among middle-aged and older adults, sedentary behavior has been associated with cardiovascular diseases, type 2 diabetes, obesity, and certain cancers [7–9]. Emerging longitudinal and meta-analytic evidence further suggests that excessive sedentary behavior independently predicts all-cause mortality, even after controlling physical activity levels [10, 11]. Beyond physical health, individuals with higher levels of sedentary behavior often experience social isolation, limitations in daily activities, and weakened physical function, which can lead to mental health problems such as depression, anxiety, and cognitive decline [3, 12, 13]. A systematic review found that mortality risk increases when sedentary behavior exceeds 7.5–9 hours per day, with a particularly pronounced rise beyond 9.5 hours [11]. Ultimately, prolonged sedentary behavior poses a substantial public health threat by simultaneously undermining physical, psychological, and social well-being and reducing overall quality of life [3, 12, 13].

**Point 2:** 2. Methods. You indicate that you followed the framework proposed by DeVellis and Thorpe, which is appropriate. Nevertheless, to meet current standards (COSMIN and good practices in scale development), several aspects should be expanded and clarified:

**Point 2-1-1:** 2.1. Participants and sampling. It is necessary to specify the inclusion and exclusion criteria more precisely, as they define the population for which the instrument can be considered valid and reliable.

- **Response:** We appreciate the reviewer’s insightful comment and fully agree that clearly defined inclusion and exclusion criteria are fundamental to establishing population validity. In the original manuscript, these criteria were described only briefly, which may have limited the clarity regarding the intended target population of the Sedentary Behavior Regulation Scale (SBRS). In response, we have substantially revised the Participants and sampling section to explicitly delineate both inclusion and exclusion criteria.
- **Line 134-141:** The participants in this study were adults aged 20–59 years residing in South Korea. A proportional stratified sampling method was used to recruit a sample reflective of the national population, based on data from the Korean National Statistical Office. Stratification variables included region, sex, and age. This strategy was adopted to enhance representativeness and reduce sampling bias, consistent with recommendations for scale development studies targeting general adult populations [20]. Within each stratum, eligible participants were randomly selected from the EMBRAIN online research panel using a computerized randomization procedure to ensure proportional allocation while maintaining random selection.
- **Line 150-158:** The inclusion criteria were adults aged 20–59 years who resided in South Korea, were able to read and understand Korean, engaged in sedentary activities as part of their daily work or leisure, and voluntarily consented to participate after receiving an explanation of the study’s purpose. Exclusion criteria included self-reported cognitive impairment, intellectual disability, severe psychiatric or neurological conditions that could interfere with comprehension or self-reporting, and inability to complete an online questionnaire independently. These criteria were specified to clearly define the target population for which the Sedentary Behavior Regulation Scale was intended to be valid and reliable, consistent with COSMIN recommendations regarding population validity [21].

**Point 2-1-2:** Please describe in greater detail the sampling/selection procedure (how strata were defined, how randomisation was performed, and how participants were assigned to the EFA and CFA). You state that you split the sample to conduct EFA and CFA, which is appropriate. However, it would be advisable to describe the splitting procedure explicitly (e.g., simple random allocation) and, if possible, consider methods that ensure equivalent subsamples on characteristics relevant to factor analysis, such as the SOLOMON method (Lorenzo-Seva, 2022), or at least report evidence of equivalence between subsamples.

- **Response:** We sincerely thank the reviewer for this detailed and methodologically important comment. In response to this comment, we have revised the Participants and sampling section to provide a more explicit description of how the strata were defined, how participants were randomly selected within strata, and how the total sample was divided into independent subsamples for EFA and CFA.
- **Line 159-166:** The required sample size for the validation process was determined based on established methodological guidelines for factor analysis. For exploratory factor analysis (EFA), a minimum ratio of 10 participants per item was applied, following the recommendations of DeVellis and Thorpe [20]. This resulted in a minimum sample size of 320 participants with 32 initial items. For confirmatory factor analysis (CFA), a sample size between 200 and 400 was considered appropriate based on the structural equation modeling guidelines [22]. Accordingly, a total target sample size of 600 participants was established to allow for independent samples for EFA and CFA.

**Point 2-1-3:** Please clarify what the EMBRAIN panel is (characteristics, coverage, quality control mechanisms, representativeness, and potential biases), as this affects external validity.

- **Response:** We thank the reviewer for this important comment regarding the EMBRAIN panel and its implications for external validity. In response, we have revised the Research participants and sampling section to provide a detailed description of the EMBRAIN online research panel.
- **Line 142-149:** Participants were recruited via EMBRAIN, a representative online research panel in South Korea consisting of a diverse pool of voluntarily registered adults. To ensure a nationally representative sample, the panel is continuously expanded and demographically balanced. To enhance data integrity, EMBRAIN implements stringent quality control measures, including identity verification, duplicate account detection, and the use of attention-check items. Furthermore, we monitored response times and excluded inconsistent or patterned responses to mitigate careless responding. Only participants who met the eligibility criteria and passed all quality control protocols were included in the final analysis.

**Point 2-2-1**: 2.2. Construct definition and item generation. Please indicate how many researchers participated in defining the construct and generating the initial item pool, and describe the consensus procedure.

- **Response:** We thank the reviewer for this important comment regarding the transparency of the construct definition and item generation process. In response, we revised the Research procedure section (Step 1 and Step 2) to explicitly describe the research team composition and the consensus-based approach used throughout these phases. We clarified that a research team of three researchers participated in defining the construct of sedentary behavior regulation and generating the initial item pool.
- **Line 182-196:** The initial step involved defining the construct of sedentary behavior regulation through a comprehensive literature review and qualitative field study. This phase was conducted by a team comprising three researchers (two nursing researchers with expertise in scale development and one psychology researcher), all of whom participated in the construct definition process. The theoretical phase involved an analysis of 24 research articles on instrument development using databases such as the Research Information Sharing Service (RISS), Korea Institute of Science and Technology Information System (KISIS), PubMed, and the Cumulative Index to Nursing and Allied Health Literature (CINAHL). Previous domestically and internationally developed tools measuring physical activity and sedentary behavior were reviewed [14, 24–32]. Each researcher independently examined the literature and proposed preliminary conceptual domains related to the regulation of sedentary behavior. Consensus on the construct definition and its core components was reached through iterative discussions until full agreement was achieved among all researchers. Through this process, self-awareness, behavioral practices, and preventive environmental design were identified as the core components of autonomous motivation for regulating sedentary behavior.
- **Line 211-220:** An item pool reflecting each component was developed through a comprehensive analysis of theoretical and qualitative findings. All three researchers independently generated candidate items corresponding to each identified component. These items were then compiled into a single pool and overlapping or redundant items were identified. A structured consensus procedure was applied, during which the research team reviewed each item for conceptual relevance, clarity, and consistency with the construct definition. Items were retained, revised, or removed based on unanimous agreement among the researchers. This consensus-based approach ensured that the initial item pool adequately represented the content domain of sedentary behavior regulation in accordance with the recommended practices for establishing content validity [20, 21].

**Point 2-2-2**: If initial interviews or qualitative data collection were conducted, please describe more clearly how that information contributed to instrument development (how it was coded, how it was transformed into items, and how traceability was ensured).

- **Response:** We thank the reviewer for this valuable comment regarding the role of qualitative data in instrument development. In response, we revised the Research procedure section (Step 1: Defining the construct) to provide a clearer and more detailed account of how qualitative interview data contributed to the development of the Sedentary Behavior Regulation Scale. We appreciate the reviewer’s suggestion, which has helped to strengthen the methodological transparency and rigor of the qualitative component of the study.
- **Line 197-207:** In the field study phase, individual interviews were conducted with eight working adults (men and women) to confirm the attributes of sedentary behavior control identified in the theoretical study. Two researchers independently reviewed the interview transcripts using a directed content analysis approach guided by the preliminary theoretical framework. Meaning units related to sedentary behavior regulation were coded and grouped into categories corresponding to the identified components. After independent coding, discrepancies were resolved through discussion until consensus was reached. The finalized qualitative categories were then compared with the theoretically derived components, and areas of convergence were used to refine construct definitions. To ensure traceability, qualitative categories served as direct sources of candidate items, allowing each item to be linked to both theoretical and empirical evidence.

**Point 2-3-1**: 2.3. Content validity. Content validity is the key measurement property for new instruments. Reporting could be strengthened as follows: In addition to the quantitative calculation of the I-CVI, it would be advisable to explicitly incorporate a qualitative evaluation by experts and/or the target population (structured comments on relevance, clarity, and comprehensiveness) and explain how disagreements were resolved.

- **Response:** We thank the reviewer for emphasizing the importance of content validity in new instrument development. In response, we revised the expert review procedure to explicitly incorporate qualitative evaluations of item relevance, clarity, and comprehensiveness in addition to the quantitative I-CVI assessment.
- **Line 228-237:** Each expert rated item relevance using a 4-point scale ranging from 1 (not at all valid) to 4 (very valid). The item content validity index (I-CVI) was calculated based on these ratings, and only items with an I-CVI of .80 or higher were retained. To adjust for chance agreement among experts, modified kappa statistics were calculated for each item based on the I-CVI and the number of experts, with values interpreted according to established benchmarks indicating substantial to excellent agreement beyond chance [33]. Scale-level content validity was further examined using the S-CVI/Ave. Quantitative indices and structured qualitative expert comments on relevance, clarity, and comprehensiveness were jointly considered and items were revised or removed through consensus as needed.

**Point 2-3-2**: 2.3. Content validity. Please consider reporting scale-level indices (e.g., S-CVI/Ave and, if applicable, S-CVI/UA), not only item-level indices.

- **Response:** We thank the reviewer for this helpful suggestion regarding content validity reporting. In response, we have added scale-level content validity indices, including the Scale-Level Content Validity Index using the averaging method (S-CVI/Ave).
- **Line 228-237:** Each expert rated item relevance using a 4-point scale ranging from 1 (not at all valid) to 4 (very valid). The item content validity index (I-CVI) was calculated based on these ratings, and only items with an I-CVI of .80 or higher were retained. To adjust for chance agreement among experts, modified kappa statistics were calculated for each item based on the I-CVI and the number of experts, with values interpreted according to established benchmarks indicating substantial to excellent agreement beyond chance [33]. Scale-level content validity was further examined using the S-CVI/Ave. Quantitative indices and structured qualitative expert comments on relevance, clarity, and comprehensiveness were jointly considered and items were revised or removed through consensus as needed.
- **Line 347-351:** Items were excluded if they demonstrated a low item-level content validity index (I-CVI ≤ .80), conceptual redundancy, or insufficient relevance to the study’s objectives (Supplementary Table 2). At this stage, the scale-level content validity index calculated using the averaging method (S-CVI/Ave) for the initial item pool was .87, indicating good overall content validity.
- **Line 352-355: After item refinement and reduction to 32 items, the S-CVI/Ave increased to .96. A second round of content validity testing was conducted with a panel of five experts to reexamine the remaining items. All items demonstrated I-CVI values of .80 or higher, leading to their final retention.**

**Point 2-3-3**: 2.3. Content validity. I suggest adding a correction for chance agreement to the I-CVI using the modified kappa (k*) (or, at minimum, justifying why it was not applied). This would strengthen the content validity argument. I attach a helpful reference:

- **Response:** We thank the reviewer for this valuable suggestion. Given the relatively large number of experts (n = 7) and the high I-CVI values observed, we followed common practice in recent scale development studies and focused on I-CVI and S-CVI/Ave. However, we now explicitly justify this decision in the manuscript and acknowledge the modified kappa as a complementary index.
- **Line 355-356: The modified kappa values ranged from .85 to .99, indicating excellent agreement beyond chance for all items.**

**Point 2-3-4**: 2.3. Content validity. It would be useful to include, as supplementary material, a table describing the experts’ characteristics (profile, experience, selection criteria) and item-by-item results (I-CVI by round, decisions: retain/modify/delete).

- **Response:** We thank the reviewer for this constructive suggestion to enhance transparency in content validity reporting. In response, we have prepared supplementary tables describing item-by-item content validity results, including I-CVI values and decisions to retain, modify, or delete items.
- **Supplementary Table 1 & Table 2**

**Point 2-4**: 2.4. Face validity. The pilot testing is appropriate. However, to align with COSMIN, it would be desirable to describe it as an evaluation of comprehensibility and, if possible, complement it with cognitive interviews (even in a small sample). If it only remains purely quantitative, please describe decision criteria (cut-offs) and the item refinement procedure.

- **Response:** We thank the reviewer for this valuable comment regarding face validity. In response, we revised the pilot testing procedure to explicitly describe it as a quantitative evaluation of item comprehensibility, in line with COSMIN recommendations. We clarified the decision criteria by specifying cut-off values for comprehensibility ratings and described how items were reviewed and refined based on these quantitative results.
- **Line 239-245:** A pilot study was conducted to quantitatively evaluate the comprehensibility of the preliminary items as an aspect of face validity. Ten adults aged 20–59 years were recruited to assess item clarity and ease of understanding. Participants rated their comprehension of each item on a 4-point scale ranging from 1 (difficult to understand) to 4 (very easy to understand). Items with mean comprehensibility scores below 3.0 were reviewed and revised to improve clarity. This quantitative decision rule was applied uniformly to all items, and only items that met the predefined comprehensibility criteria were retained for subsequent large-scale administrations.

**Point 2-5-1**: 2.5. Psychometric analysis (EFA/CFA) and reliability. Please clarify whether the factor analysis was based on Pearson or polychoric correlations, and justify the decision given the Likert format. It should be stated in the Methods that KMO and Bartlett’s test were used to assess sampling adequacy. Please specify the extraction method, the factor retention criteria (I would recommend including parallel analysis and/or a scree plot, beyond the eigenvalue > 1 criterion), and the criteria used for cross-loadings.

- **Response:** We thank the reviewer for these valuable methodological suggestions regarding factor analysis. In response, we have clarified that factor analyses were conducted based on Pearson correlation matrices, and we provided a justification for this decision given the use of a 5-point Likert scale and the acceptable distributional properties of the items. We also explicitly stated the use of the Kaiser–Meyer–Olkin (KMO) measure and Bartlett’s test of sphericity to assess sampling adequacy. In addition, we expanded the description of factor retention criteria by including scree plot inspection alongside the eigenvalue > 1 criterion and clarified the criteria used to identify and remove cross-loading items.
- **Line 311-319:** Construct validity was evaluated using a two-step factor analytic approach. Prior to factor analyses, chi-square tests were conducted to examine the equivalence between the EFA (n = 320) and CFA (n = 280) groups, confirming the homogeneity of the two subsamples. EFA was conducted using Pearson correlation matrices, as the items were measured on a 5-point Likert scale and demonstrated acceptable normality based on skewness and kurtosis values. A maximum likelihood extraction method with Promax rotation was applied. Sampling adequacy was assessed using the Kaiser–Meyer–Olkin (KMO) measure and Bartlett’s test of sphericity prior to factor extraction. Factor retention decisions were informed by multiple criteria, including eigenvalues greater than 1.0 and inspection of the scree plot.
- **Line 383-378:** An EFA was performed on the remaining 25 items to examine the underlying factor structure. The suitability of the data for factor analysis was confirmed using a KMO value of .93 and a statistically significant Bartlett’s test of sphericity (χ^2^ = 3831.94, df = 300, *p* < .001). Both eigenvalues greater than 1.0 and scree plot inspection supported a two-factor solution. During the analysis, four items (x8, x19, x23, and x24) were sequentially removed because their communality values were below .30. An additional five items (x13, x17, x21, x25, and x30) were removed because of cross-loadings, defined as factor loadings of .40 or higher on two factors in the pattern matrix.

**Point 2-5-2**: 2.5. Psychometric analysis (EFA/CFA) and reliability. For reliability, Cronbach’s α is commonly used but insufficient as the sole indicator; I recommend adding ω (omega) and justifying its use based on the final structure. Please review page 49 of: DeVellis RF, Thorpe CT. *Scale development: Theory and applications* (5th ed.). Sage, where the limitations of Cronbach’s alpha are discussed.

- **Response:** We thank the reviewer for highlighting the limitations of relying solely on Cronbach’s α to assess reliability. In response, we supplemented Cronbach’s α with McDonald’s omega (ω), which provides a more appropriate estimate of internal consistency for congeneric measures and multidimensional factor structures.
- **Line 329-331:** Reliability was evaluated using Cronbach’s α and McDonald’s omega (ω) for internal consistency, as well as the intraclass correlation coefficient (ICC) for test–retest reliability.
- **Line 452-456:** Cronbach’s α for the entire instrument was .87, indicating high internal consistency. Factor 1 had a value of .83, and Factor 2 had a value of .82, both of which exceeded the recommended benchmark of .80. The McDonald’s omega (ω) coefficient for the entire instrument was .87, while Factor 1 and Factor 2 showed ω values of .81 and .82, respectively, indicating satisfactory internal consistency under a congeneric measurement model.

**Point 2-5-3**: 2.5. Psychometric analysis (EFA/CFA) and reliability. When reporting CFA fit indices, please indicate the cut-off values used to define an acceptable/good model and avoid purely data-driven modifications unless they are theoretically justified. In addition, confidence intervals were not calculated for all estimates, which reduces the quality of the validation evidence. Please consider this aspect in future studies.

- **Response:** We sincerely appreciate your careful attention to detail. We have clarified the cut-off values used to define acceptable and good CFA model fit based on widely accepted guidelines and explicitly stated that model modifications were guided by theoretical considerations rather than purely data-driven criteria.
- **Line 405-410:** CFA was conducted to examine the structural relationships between the two factors derived from EFA. Model fit was evaluated using established cut-off criteria: χ²/df values below 3.0, CFI and TLI values ≥ .90, SRMR ≤ .08, and RMSEA ≤ .08 were considered indicative of acceptable model fit, while lower χ²/df and RMSEA values and higher CFI and TLI values indicated good fit. The initial CFA model demonstrated suboptimal fit, with a χ^2^/df value of 3.43, and other indices that did not meet recommended standards (CFI = .88, TLI = .86, SRMR = .084, RMSEA = .093, 90% CI [.083, .104]).

**Point 2-6**: 2.6. Criterion validity. If an external instrument is used as a criterion, please provide more information on its scoring system and its psychometric properties in the linguistic/cultural context of application, so that readers can interpret the magnitude and relevance of the correlations. Therefore, please provide validity and reliability data for the Korean version of the GPAQ.

- **Response:** We thank the reviewer for requesting additional information regarding the external criterion. We have revised the manuscript to provide a detailed description of the scoring system and psychometric properties of the Korean version of the GPAQ, including prior evidence of its reliability and validity in Korean adults, to facilitate interpretation of the observed correlations.
- **Line 272-276:** The K-GPAQ has demonstrated acceptable test–retest reliability and criterion-related validity among Korean adults, with prior validation studies reporting moderate to good reliability across physical activity domains (Spearman’s r = .27–.70; κ = .30–.67) and significant, albeit weak, correlations with accelerometer-measured physical activity and sedentary behavior (r = .18–.34) [27].

**Point 3**: 3. Results. Some additional or expanded results could be presented as supplementary material (e.g., I-CVI per item and per round; item refinement decisions; response distributions in the pilot test). There are no data on item scores in the pilot test, and these could be provided as supplementary material. I also await the editor's opinion on this matter.

- **Response:** We thank the reviewer for the valuable suggestion regarding the presentation of additional results. In response, we have added supplementary tables providing detailed information on the item development process, including item-level I-CVI values by round, and item refinement decisions (retain/modify/delete). We also appreciate the reviewer’s note and will follow the editor’s guidance regarding the extent of supplementary material.
- **Supplementary Table 2.**

**Point 4**: 4. Presentation and interpretation of the final instrument. Please clarify precisely the scoring method (total score vs. subscales; minimum–maximum ranges; interpretation of higher scores) and whether any proposed cut-offs exist or whether interpretation is only relative.

- **Response:** We thank the reviewer for requesting clarification regarding the scoring and interpretation of the final instrument. In response, we have clarified that no empirical cut-off scores are proposed at this stage and that interpretation of the SBRS is intended to be relative rather than categorical, given the developmental nature of the scale.
- **Line 461-470:** This study finalized the SBRS, a 12-item instrument that assessed an individual’s ability to manage sedentary behaviors. The items are structured on a 5-point Likert scale ranging from 1 (strongly disagree) to 5 (strongly agree), with higher scores indicating more effective sedentary behavior management. The total SBRS score was calculated by summing all 12 items, yielding a possible range of 12–60. The final item composition by sub-factor was as follows: Factor 1 (sedentary behavior management and environmental support) comprised items 1–6, and Factor 2 (active movement in sedentary contexts) comprised items 7–12 (Appendix 1). At the current stage of scale development, no empirical cut-off scores have been proposed; thus, SBRS scores are intended to be interpreted relative to sample distributions or for comparative purposes across individuals, groups, or time points.

**Point 5**: I believe these comments are sufficient for a first round. Overall, I consider your results promising, but the manuscript requires substantial improvement in methodological reporting and, in particular, in the rationale and documentation of content validity. I hope these observations are helpful in strengthening your work and are received as an opportunity for improvement.

- **Response:** We sincerely thank the reviewer for the thorough and constructive evaluation of our manuscript. We greatly appreciate the recognition of the promise of our results and the insightful comments regarding methodological reporting and the documentation of content validity. We have carefully addressed all suggestions and revised the manuscript accordingly, and we view these comments as a valuable opportunity to strengthen the rigor, clarity, and overall quality of our work.

**Reviewer 2’s comment**

**Point 1-1:** 1. Insufficient Convergent Validity (AVE). The average variance extracted (AVE) values for both factors are below the recommended threshold of 0.50, indicating insufficient convergent validity. Although the authors acknowledge this limitation, it remains a serious concern regarding the validity of the scale. A low AVE means that the variance of the observed variable is due to more Error than the latent variable. Therefore, there is a risk that using this measure in future studies will not provide sufficient statistical power. If the AVE is low, the discriminant validity between factors (Fornell-Larcker criteria or HTMT ratio) may also not be met.

- **Response:** We acknowledge the reviewer’s concern regarding the AVE values below .50. We appreciate the reviewer’s concern regarding the low AVE values and their potential implications for discriminant validity. In response, we conducted an additional discriminant validity assessment using the heterotrait–monotrait ratio (HTMT). The HTMT value between the two factors was .69, which is well below the conservative cut-off value of .85, indicating adequate discriminant validity despite the relatively high inter-factor correlation (r = .68).
- **Line 322-327:** Convergent validity was confirmed by examining correlations, construct reliability (CR), and average variance extracted (AVE) among the sub-factors. Discriminant validity was evaluated using the Fornell–Larcker criterion [34] and the heterotrait–monotrait (HTMT) ratio. HTMT values were computed based on item-level correlations, with values below the .85 threshold considered indicative of adequate discriminant validity [35].
- **Line 422-432:** Reliability was confirmed, as both factors showed CR values above .70. However, the AVE was below the recommended .50 benchmark for both factors, indicating insufficient convergent validity. This finding suggests that future instrument refinement is required to enhance this aspect. Discriminant validity was examined using the Fornell–Larcker criterion. The square root of AVE for Factor 1 (.67) was slightly lower than the inter-factor correlation (r = .68, *p* < .001), while the value for Factor 2 (.68) was equivalent to the correlation, indicating limited to marginal discriminant validity based on this criterion alone. To further evaluate discriminant validity, the HTMT value was calculated. The HTMT value between Factors 1 and 2 was .69, well below the conservative threshold of .85. These results indicate that although the two factors are moderately correlated, they represent empirically distinct constructs.

**Point 1-2**: The authors are encouraged to reconsider the current items and, if possible, collect additional data to refine the scale. Alternatively, the study could be explicitly positioned as a preliminary or developmental study, with a stronger emphasis on the need for further refinement and validation. In the Discussion section, the authors should elaborate more clearly on how insufficient AVE may affect the interpretation and application of the SBRS in research and practice.

- **Response:** We appreciate the reviewer’s thoughtful suggestion regarding further refinement of the SBRS. In response, we have explicitly positioned the present study as a developmental validation study and expanded the Discussion to clarify how insufficient AVE may affect the interpretation and application of the scale. We also emphasized the need for additional data collection and item refinement in future research to enhance variance extraction and strengthen construct validity.
- **Line 575-586:** Fourth, the AVE values for both factors did not reach the recommended threshold of .50, indicating that a substantial proportion of the variance in the observed items may be attributable to measurement error rather than latent constructs. Although discriminant validity was supported by the HTMT criterion, suggesting that the two factors are empirically distinct, insufficient AVE may limit construct precision and reduce statistical power in subsequent analyses. In particular, associations between SBRS scores and related outcomes may be underestimated, and caution is warranted when interpreting small effect sizes or non-significant findings. Taken together, these limitations indicate that the present study represents an initial, developmental validation aimed at establishing the initial psychometric properties of the SBRS rather than a definitive validation. These findings highlight the need for further refinement of item content and factor structure to enhance variance extraction and measurement precision.
- **Line 594-597:** Third, additional data should be collected to refine and revalidate the current item set, with particular emphasis on revising or replacing items with lower factor loadings to improve convergent validity and AVE. Such refinement is expected to enhance the sensitivity and statistical power of the scale in future research applications.

**Point 2-1**: 2. Insufficient Theoretical Justification for the Factor Structure. In the conceptual framework, three core components—self-awareness, behavioral practice, and preventive environmental design—are identified as key elements of sedentary behavior regulation. However, the final validated scale consists of a two-factor structure, and all items related to self-awareness were excluded during the factor analytic process. Although this discrepancy is briefly mentioned in the Discussion, the theoretical justification is insufficient. A deeper discussion is needed to explain why self-awareness, which was theoretically emphasized, failed to emerge as a statistically meaningful factor, and what this implies for the conceptualization of sedentary behavior regulation. In addition, while the SBRS is described as being grounded in Self-Determination Theory (SDT), it remains unclear how the two final factors—“sedentary behavior management and environmental support” and “active movement in sedentary contexts”—correspond to the three basic psychological needs proposed by SDT (autonomy, competence, and relatedness). A clearer and more systematic explanation of this theoretical alignment is necessary.

- **Response:** We sincerely thank the reviewer for highlighting the need for stronger theoretical justification regarding the final factor structure. In response, we substantially expanded the Discussion and conceptual framework to explain why self-awareness, although theoretically important, did not emerge as an independent factor and may function as a cognitive antecedent rather than an enacted regulatory dimension.
- **Line 510-526:** This study conceptualized three core components—self-awareness, behavioral practice, and preventive environmental support—based on SDT to develop a sitting behavior regulation scale. An important theoretical implication of this study is the exclusion of self-awareness items during the factor analytic process. Although self-awareness was initially conceptualized as a core component of sedentary behavior regulation, all related items were removed because of insufficient psychometric performance. This finding suggests that self-awareness may function as a cognitive antecedent or prerequisite for regulation rather than as an independent regulatory dimension. Given the habitual and often unconscious nature of sedentary behavior, awareness alone may be insufficient to produce meaningful behavioral changes unless translated into concrete actions and environmental modifications. Accordingly, the final two-factor structure indicates that behavioral practice and environmental support constitute the most salient and measurable dimensions of sedentary behavior regulation. This interpretation is consistent with SDT, which emphasizes enacted, self-determined behavior as the primary mechanism through which motivation is translated into sustained behavioral change. From an intervention perspective, these findings imply that programs aimed at reducing sedentary behavior may be more effective if they prioritize actionable strategies and supportive environments over raising awareness.

**Point 2-2**: In addition, while the SBRS is described as being grounded in Self-Determination Theory (SDT), it remains unclear how the two final factors—“sedentary behavior management and environmental support” and “active movement in sedentary contexts”—correspond to the three basic psychological needs proposed by SDT (autonomy, competence, and relatedness). A clearer and more systematic explanation of this theoretical alignment is necessary. Scale development should be a consistent process: theory (SDT), item generation (32 questions), statistical examination (EFA/CFA), and final scale (12 questions). However, the current situation seems to separate theory from the final statistical results. In particular, please consider the theoretical implications of the omission of self-awareness.

- **Response:** We thank the reviewer for the constructive comments regarding the theoretical coherence of the factor structure. In response, we substantially reorganized and expanded the Discussion to provide a systematic interpretation linking Self-Determination Theory, the item development process, and the final two-factor structure, with particular emphasis on the theoretical implications of the omission of self-awareness.
- **Line 110-126:** Grounded in the principles of Self-Determination Theory (SDT), this study was designed to develop a new Sedentary Behavior Regulation Scale (SBRS). SDT is a prominent motivation theory that emphasizes the importance of internal psychological resources in personality development and behavioral regulation [17, 18]. This theory posits that when basic psychological needs (competence, autonomy, and relatedness) are satisfied, intrinsic motivation is enhanced, thereby facilitating long-term behavioral change and positively influencing outcomes [14, 17, 19]. Consistent with this framework, sedentary behavior regulation is conceptualized as an autonomously motivated process involving self-awareness of sedentary patterns, active behavioral practices to interrupt or reduce sedentary time, and preventive environmental structuring to minimize prolonged sitting. Accordingly, SBRS is theoretically defined as an individual’s conscious and active process of managing sedentary behavior in daily life. Operationally, this construct is represented by the total score obtained from the developed scale, with higher scores indicating a greater capacity for effective sedentary behavior regulation. Within this framework, self-awareness is conceptualized not as an independent regulatory dimension but as a cognitive prerequisite that enables—rather than constitutes—the enactment of sedentary behavior regulation through behavioral practices and environmental structuring.
- **Line 510-526:** This study conceptualized three core components—self-awareness, behavioral practice, and preventive environmental support—based on SDT to develop a sitting behavior regulation scale. An important theoretical implication of this study is the exclusion of self-awareness items during the factor analytic process. Although self-awareness was initially conceptualized as a core component of sedentary behavior regulation, all related items were removed because of insufficient psychometric performance. This finding suggests that self-awareness may function as a cognitive antecedent or prerequisite for regulation rather than as an independent regulatory dimension. Given the habitual and often unconscious nature of sedentary behavior, awareness alone may be insufficient to produce meaningful behavioral changes unless translated into concrete actions and environmental modifications. Accordingly, the final two-factor structure indicates that behavioral practice and environmental support constitute the most salient and measurable dimensions of sedentary behavior regulation. This interpretation is consistent with SDT, which emphasizes enacted, self-determined behavior as the primary mechanism through which motivation is translated into sustained behavioral change. From an intervention perspective, these findings imply that programs aimed at reducing sedentary behavior may be more effective if they prioritize actionable strategies and supportive environments over raising awareness.

**Point 3**: 3. Insufficient Explanation of the Item Reduction Process. During the reduction from 32 initial items to the final 12-item scale, a total of 20 items were removed (9 during EFA and an additional 4 during CFA). However, the manuscript does not provide sufficient detail regarding the content of the deleted items or the specific reasons for their removal. The authors should provide a list of the excluded items and clearly explain the rationale for deletion. In particular, items described as having “redundant content” require more concrete and transparent justification.

- **Response:** We thank the reviewer for pointing out the need for greater transparency in the item reduction process. In response, we have added a detailed description of the excluded items and the specific reasons for their removal during both EFA and CFA, including low communalities, cross-loadings, and conceptual redundancy.
- **Line 379-381:** As a result of the item-to-total correlation analysis, seven items with a correlation coefficient (r) of less than .30 were removed because of their low discriminatory power, resulting in the selection of 25 items for further analysis.
- **Line 386-390:** During the analysis, four items (x8, x19, x23, and x24) were sequentially removed because their communality values were below .30. An additional five items (x13, x17, x21, x25, and x30) were removed because of cross-loadings, defined as factor loadings of .40 or higher on two factors in the pattern matrix.
- **Line 411-415:** To avoid purely data-driven modifications, an iterative model refinement process was conducted based on modification indices (MI) and theoretical considerations, including conceptual overlap and redundancy among items with highly similar behavioral intent. This process led to the deletion of four items (x28, x31, x15, and x18), resulting in a final model with 12 items (Figure 1).

**Point 4-1**: 4. Issues Related to Sample Size and Sample Splitting. Although the rationale for the initial sample size calculation is described, the subsequent reduction from 32 to 12 items raises concerns about whether the original justification remains appropriate. This issue should be addressed explicitly. Furthermore, regarding the division of the sample into EFA (n = 320) and CFA (n = 280) groups, it is unclear whether the split was fully random and whether key demographic characteristics (e.g., age, sex) were equivalent between the two groups. This information should be clearly reported.

- **Response:** We appreciate the reviewer’s careful attention to issues related to sample size and sample splitting. In response, we have clarified that the initial sample size was determined conservatively to satisfy recommended requirements for both EFA and CFA based on the initial 32-item pool, and that the final sample sizes for EFA (n = 320) and CFA (n = 280) remain adequate for the reduced 12-item model according to established guidelines. We have also explicitly described the random allocation procedure used to split the sample and added a comparison of key demographic characteristics (age and sex) between the EFA and CFA groups, confirming that the two subsamples were statistically equivalent.
- **Line 364-370:** There were 600 survey participants, 312 (52.03%) men and 288 (48.0%) women. The largest age group were those in their 50s (n = 182; 30.3%). To establish construct validity, the total sample was randomly divided into two independent subsamples for EFA (n=320) and CFA (n=280). To verify subsample comparability, main demographic characteristics, including age and sex, were examined between the EFA and CFA groups. No statistically significant differences were found, indicating that the two subsamples were demographically equivalent and suitable for independent factor analytic procedures (Table 1).

**Point 4-2**: In addition, the restriction of the sample to Korean adults aged 20–59 years should be more explicitly discussed as a limitation affecting the generalizability of the findings.

- **Response:** We appreciate the reviewer’s comment regarding the age restriction of the sample. In response, we have revised the Limitations section to more explicitly acknowledge that restricting the sample to Korean adults aged 20–59 years limits the generalizability of the findings to older adults and other cultural contexts. We also emphasized the need for future validation studies in more diverse age groups and populations.
- **Line 570-575:** Third, the sample was restricted to Korean adults aged 20–59 years, which limits the generalizability of the findings to older adults, particularly those aged 60 years and older, who may exhibit distinct sedentary behavior patterns, health conditions, and regulatory processes. In addition, cultural specificity should be considered, as sedentary behavior and its regulation may vary across sociocultural contexts. Accordingly, future studies should validate the SBRS across diverse age groups and populations.

**Point 5**: 5. Interpretation of Effect Sizes for Criterion Validity. The correlation between the SBRS and physical activity (r = .19) is statistically significant but represents a small effect size, while the correlation with sedentary time (r = −.33) is moderate. On this basis, concluding that the scale demonstrates “strong criterion validity” appears to be an overstatement. The authors should provide a clearer theoretical rationale explaining why correlations of this magnitude are considered appropriate or meaningful for a measure of sedentary behavior regulation, and the interpretation should be more cautious.

- **Response:** We thank the reviewer for the careful evaluation of the effect sizes related to criterion validity. In response, we have revised the interpretation to avoid overstating the strength of the associations and clarified that the observed correlations represent small to moderate effect sizes.
- **Line 33-35:** Criterion-related validity was supported by a significant positive correlation with physical activity (r = .19, *p* < .001) and a significant negative correlation with sedentary time (r = −.33, *p* < .001).
- **Line 549-552:** The SBRS showed a statistically significant positive correlation with physical activity and a negative correlation with sedentary time. Although these effect sizes range from small to moderate, they are theoretically meaningful given that the SBRS is designed to assess regulatory capacity rather than direct behavioral frequency.

**Point 6**: 6. Insufficient Description of Statistical Analyses. Although the estimation method used for structural equation modeling is reported, the manuscript does not specify how missing data were handled. In addition, the authors should report whether and how the assumptions of normality were examined and whether the data met these assumptions.

- **Response:** We thank the reviewer for highlighting the need for a clearer description of the statistical assumptions and missing data handling. In response, we have clarified that cases with missing data were excluded prior to analysis and that all analyses were conducted using complete cases only. We have also added a detailed description of how univariate normality was examined using skewness and kurtosis statistics, confirming that the data met the assumptions required for maximum likelihood estimation.
- **Line 302-310:** Data analysis was conducted using the SPSS 26.0 and AMOS 23.0 software (IBM Corp., Armonk, NY, USA). Prior to analysis, incomplete responses were removed, and only complete responses were included in EFA, CFA, and reliability analysis. Descriptive statistics were calculated for participants’ general characteristics. Item analysis involved checking the means, standard deviations, skewness, and kurtosis values of the initial items. To assess the assumption of normality required for maximum likelihood estimation, univariate normality was evaluated by inspecting skewness and kurtosis values for all items, which fell within acceptable ranges. Correlation coefficients between the total score and each item were calculated, and items with correlations below .30 were examined.

**Point 7**: 7. Insufficient Evidence for Cultural Validity. The manuscript refers to the Korean floor-sitting culture as an important contextual factor; however, it is unclear how the final 12 items specifically reflect this cultural characteristic. Additional explanation or examples are needed to justify the cultural relevance of the developed scale.

- **Response:** We thank the reviewer for highlighting the need for clearer evidence of cultural validity. In response, we have expanded the Discussion to explicitly explain how the final SBRS items capture regulatory behaviors that are particularly relevant in the Korean context, including frequent floor-sitting, posture adjustment, and movement within seated activities.
- **Line 527-539:** Although the final 12 items of the SBRS do not explicitly reference floor sitting, they were intentionally designed to capture regulatory behaviors that are prevalent in sedentary contexts characteristic of Korean daily life. In Korea, sedentary behavior often occurs not only in chair-based settings but also in floor-seated postures during activities such as dining, leisure, and social interaction. Items addressing frequent postural adjustment, stretching, standing up in response to discomfort, and intentional movement during prolonged sitting reflect self-regulatory strategies commonly used to mitigate the physical strain associated with floor-based and low-seated positions. Accordingly, the SBRS captures culturally embedded patterns of sedentary behavior regulation by focusing on how individuals manage bodily discomfort and initiate movement within seated contexts rather than restricting assessment to chair-based sedentary time. This approach preserves cultural sensitivity while maintaining conceptual generalizability, supporting applicability within the Korean sociocultural context and potential adaptability to other cultures with similar sedentary practices.

**Point 8**: 8. Inconsistencies in Terminology and Language Issues. There are several inconsistencies and language-related issues throughout the manuscript, including: Inconsistent use of “sedentary behavior regulation”, “sedentary behavior management”, and “sedentary behaviour regulation”. Inconsistent use of “subfactor” and “sub-factor”. Inconsistent use of “hour” and “hours”. In addition, some sentences are verbose or grammatically awkward. Professional English language editing is strongly recommended.

- **Response:** We thank the reviewer for pointing out the inconsistencies in terminology and language usage throughout the manuscript. In response, we have carefully revised the manuscript to ensure consistent use of key terms. In addition, the manuscript has undergone professional English language editing to improve clarity, conciseness, and grammatical accuracy.
- We attach a certificate for professional English proofreading. (File name: Certificate_of_editing-SYURE)

**Point 9-1**: 9. Other Issues. Please verify the accuracy of the data collection period and the timing of ethical approval.

- **Response:** We thank the reviewer for requesting clarification regarding the timing of ethical approval and data collection. Ethical approval was obtained from the Institutional Review Board in March 2025, and data collection was subsequently conducted between July 17 and July 22, 2025. We have carefully verified and confirmed that the data collection period occurred after IRB approval and have ensured that this information is reported accurately and consistently in the manuscript.
- **Line 247-250:** Data was collected through an online panel survey conducted by EMBRAIN Co., Ltd. (Seoul, Republic of Korea), a company specializing in online surveys. From July 17 to July 22, 2025, invitation emails were randomly sent to 7,977 of the company’s 1.78 million panelists using a sampling method based on population proportionality.
- **Line 286-288:** This study was conducted in accordance with ethical guidelines and was approved by the Institutional Review Board (IRB No: SYU 2025-03-030-001**)** in March 2025, prior to data collection.

**Point 9-2**: In Table 1, the CFA value for Residential area: Gyeonggi-do appears to be incorrect and should be carefully checked.

- **Response:** We would like to thank the reviewer who carefully pointed out a potential issue with the CFA values for the residential area category (Gyeonggi Province) in Table 1. We have rechecked the original data set and corrected the values accordingly.
- **Line 372-373:** Table 1. General Characteristics of the Participants (N=600)
