# Supplementary material for: Development and validation of the sedentary behavior regulation scale in Korean Adults Population
Source: PLoS One. 2026 Apr 13;21(4):e0346963. doi: 10.1371/journal.pone.0346963 (PMC13075669; doi:10.1371/journal.pone.0346963)
Supplement: Attachment — Submitted filename: Response to reviewer_0325.docx [file pone.0346963.s007.docx]

**Dea****r Editor and Reviewers,**

We wish to thank you for your thoughtful comments and valuable feedback on the manuscript originally titled, “**Development and Validation of the Sedentary Behavior Regulation Scale in Korean Adults Population**”.

We have modified the manuscript according to your suggestions, rewriting and rephrasing sections to improve clarity, adding further information, and explaining in detail the points that were previously vague. For your convenience, we have set the revisions in the manuscript in red. We believe that the revised version of this paper will interest the readership of the PLOS ONE.

We have seriously considered the reviewers’ comments and carefully revised the manuscript. Please find our responses to the reviewer’s comments point by point below.

-----------------------------------------------------------------------------------------------------

**Additional Editor Comments**

**Point 1:** The authors did a significant research effort to address this research gap in the Korean population. It would be more beneficial for the targeted population if this fact was explicitly stated in the title of this research by adding the phrase “in Korean adults population”.

- **Response:** Thank you for your insightful comment. We agree that explicitly specifying the target population in the title would enhance the clarity and applicability of the manuscript for readers. In response to this suggestion, we have revised the title by adding the phrase “in Korean adult population” so that the title more clearly reflects the population for which the scale was developed and validated
- **Title: Development and Validation of the Sedentary Behavior Regulation Scale in Korean Adults Population**

**Point 2:** The Moreover, the introduction of this manuscript lacks a more detailed reflection on how this self-report scale would meet the meeds of the Korean population and help them to solve this problem.

- **Response:** Thank you for your valuable comment. We agree that the original Introduction did not sufficiently explain how this self-report scale would meet the needs of the Korean population and help address sedentary behavior in Korea. Accordingly, we revised this section by adding prior research evidence on the characteristics and health risks of sedentary behavior among Korean adults, as well as culturally relevant sitting contexts in Korea. In addition, we clarified that this scale was developed to assess not only the amount of sedentary time but also the individual’s capacity to regulate and interrupt sedentary behavior in everyday life.
- **Line 97-102:** In the Korean context, where prolonged sitting is especially concentrated among office workers and other sedentary occupational groups and is associated with elevated metabolic risk, a culturally sensitive self-report scale that assesses not only sedentary duration but also the ability to interrupt and regulate sitting may provide a practical basis for identifying high-risk groups and developing tailored behavioral interventions for everyday and workplace settings [17, 18].

**Reviewer 1’s comment**

**Point 1:** Line 238: “reliability was evaluated by assessing internal consistency with Cronbach’s α and test-retest reliability using a two-week interval with a subsample of 30 adults.” Please add that McDonald’s omega was also calculated.

- **Response:** Thank you for your valuable suggestion. We have revised the sentence to explicitly indicate that McDonald’s omega was also calculated as an index of internal consistency, together with Cronbach’s α.
- **Line 260-262:** Reliability was evaluated by assessing internal consistency using Cronbach’s α and McDonald’s omega, as well as test–retest reliability over a two-week interval with a subsample of 30 adults.

**Point 2:** Decision to remove five items: You state that “An additional five items (x13, x17, x21, x25, and x30) were removed because of cross-loadings, defined as factor loadings of .40 or higher on two factors in the pattern matrix.” I respect this decision; however, for future studies, rather than removing such items, it may be more appropriate to re-evaluate them in relation to the theoretical framework and the measurement model. Cross-loadings are not necessarily indicative of poor item performance and may sometimes reflect phenomena such as essential unidimensionality. In future work, you may wish to consider assigning the item to the factor on which it loads most strongly, or to the dimension that best fits the item content based on the theoretical framework.

- **Response:** Thank you for this valuable comment. We agree that cross-loadings do not necessarily indicate poor item performance and should not be interpreted solely on statistical grounds. In response, we have added this point to the Discussion and noted that future studies should re-examine these items using theory-driven judgment and alternative modeling approaches.
- **Line 611-613:** Furthermore, items removed because of cross-loadings should be re-evaluated in future studies with closer attention to their theoretical relevance and substantive content, rather than being excluded solely on statistical grounds.

**Reviewer 2’s comment**

**Point 1:**  **1. Convergent Validity (AVE).** The addition of the HTMT analysis is a valuable supplement, and I appreciate the repositioning of the study as a "developmental validation". However, the Fornell–Larcker criterion is clearly not met for Factor 1 (√AVE = .67 < inter-factor r = .68), and only marginally met for Factor 2 (√AVE = .68 = r = .68). The current manuscript states this indicates "limited to marginal discriminant validity based on this criterion alone" (Lines 426–429), then pivots to the HTMT result as if it resolves the issue. This framing risks understating the concern. The authors should: (a) Explicitly acknowledge that the Fornell–Larcker criterion was not satisfied, rather than using softened language such as "limited to marginal." (b) In the Discussion/Limitations, note that the failure to meet the Fornell–Larcker criterion, combined with AVE < .50, raises the possibility that the two factors may not be sufficiently distinct at the construct level, and that this should be a priority for future refinement.

- **Response:** Thank you for your important comment. We have carefully revised the Discussion and Limitations sections to explicitly reflect this concern. In particular, we now acknowledge that the unmet Fornell–Larcker criterion and AVE values below .50 raise questions regarding construct-level distinctiveness, and we state that this issue should be prioritized in future refinement of the scale.
- **Line 431-434:** The Fornell–Larcker criterion for discriminant validity was not satisfied, as the square root of AVE for Factor 1 (.67) was lower than the inter-factor correlation (r = .68, p < .001), and the value for Factor 2 (.68) was only equal to the correlation.
- **Line 600-607:** Although the HTMT result suggested that the two factors may be empirically distinguishable, insufficient AVE may limit construct precision and reduce statistical power in subsequent analyses. In particular, associations between SBRS scores and related outcomes may be underestimated, and caution is warranted when interpreting small effect sizes or non-significant findings. Moreover, the failure to satisfy the Fornell–Larcker criterion, together with AVE values below .50, raises the possibility that the two factors may not yet be sufficiently distinct at the construct level, and this issue should be prioritized in future refinement of the scale.

**Point 2-1**: **2. Theoretical Justification for the Factor Structure.** The expanded discussion of self-awareness as a "cognitive antecedent or prerequisite" (Lines 510–526) is an improvement. However, two concerns remain: (a) Post-hoc modification to the conceptual framework: The revised Introduction now states that "self-awareness is conceptualized not as an independent regulatory dimension but as a cognitive prerequisite that enables—rather than constitutes—the enactment of sedentary behavior regulation" (Lines 123–126). However, this reconceptualization appears to have been introduced after the factor analysis revealed that self-awareness items performed poorly. In the original conceptual framework, self-awareness was clearly positioned as one of three core components. Presenting this post-hoc reinterpretation in the Introduction as if it were part of the original theoretical framework is misleading. The authors should either: remove this reframing from the Introduction and instead present it as a theoretical insight derived from the empirical findings in the Discussion, or clearly state in the Introduction that this conceptualization was refined based on empirical evidence from the present study.

- **Response:** Thank you for your thoughtful comment. We agree that presenting the revised role of self-awareness in the Introduction risked suggesting that this interpretation had been part of the original conceptual framework. To address this concern, we removed the corresponding statement from the Introduction and instead elaborated on this point in the Discussion. We present it as a theoretical insight derived from the empirical findings of the present study, rather than as an a priori conceptual assumption.
- **Line 519-526:** This study conceptualized three core components—self-awareness, behavioral practice, and preventive environmental support—based on SDT to develop a sitting behavior regulation scale. An important theoretical implication of this study is the exclusion of self-awareness items during the factor analytic process. This interpretation was refined on the basis of the empirical findings of the present study, rather than being assumed as part of the initial conceptual framework. Although self-awareness was initially conceptualized as a core component of sedentary behavior regulation, all related items were removed because of insufficient psychometric performance.

**Point 2-1**: **2. Theoretical Justification for the Factor Structure.** (b) Absence of "relatedness" in SDT alignment: The Discussion (Lines 491–509) explains how the two factors align with autonomy and competence, but the third basic psychological need in SDT relatedness is not addressed. Since the SBRS is explicitly grounded in SDT, the authors should discuss why relatedness does not appear to be captured by the final scale and what implications this has for the theoretical completeness of the instrument. Even a brief acknowledgment (e.g., that the individual nature of sedentary behavior regulation may explain the limited role of relatedness) would strengthen the argument.

- **Response:** We revised the Discussion to clarify that the retained factors primarily reflect the autonomy and competence dimensions of SDT, while relatedness was not clearly captured in the final scale. We also acknowledge that this may limit the theoretical completeness of the instrument regarding the full SDT framework. Consequently, we suggested that future research examine whether relatedness-based elements should be incorporated in subsequent refinements of the scale.
- **Line 513-518:** In contrast, relatedness was not clearly captured in the final scale. This may be because sedentary behavior regulation is often enacted as an individual, self-directed process rather than through interpersonal interaction. Therefore, although the SBRS reflects the autonomy and competence dimensions of SDT, future refinement may be needed to examine whether relatedness-based aspects of regulation should also be incorporated.

**Point 3**: **3. Item Reduction Process.** The manuscript now provides somewhat more detail on why items were removed during EFA (low communalities, cross-loadings) and CFA (modification indices, conceptual redundancy). However, my original request was for a list of the excluded items and their content. I understand that the authors reference Supplementary Table 2 as containing content validity and item reduction information. However: (a) The specific content (text) of the 20 removed items and the component to which each item originally belonged (self-awareness, behavioral practice, or environmental design) should be available to reviewers and readers, either in the main text or as supplementary material. Without this information, it is impossible for readers to independently evaluate whether the removed items were appropriately excluded or whether important content was lost. (b) In particular, since all self-awareness items were excluded, it would be informative to present these items and their psychometric performance (e.g., communalities, factor loadings) to support the claim that their removal was empirically justified.

- **Response:** Thank you for your important and constructive comment. We added Supplementary Table 3, which presents the excluded self-awareness items, their original conceptual classification, and their psychometric indicators in the item–total correlation coefficients exploratory factor analysis, including communalities and factor loadings. We also revised the manuscript to direct readers to this supplementary table.
- **Line 383-385:** In particular, the self-awareness items were removed because their item–total correlation coefficients were .30 or lower, indicating insufficient psychometric performance for retention in the final factor structure.
- **Line 390-396:** During the analysis, two items (x8 and x19) were removed because their communality values were below .30. An additional four items (x17, x23, x24, and x25) were removed because their factor loadings were below .40, and three items (x13, x21, and x30) were removed because of cross-loadings, defined as factor loadings of .30 or higher on two factors in the pattern matrix. The content and psychometric properties of the excluded items, including item–total correlation coefficients, communalities, and factor loadings, are presented in Supplementary Table 3.
- **Supplementary Table 3. Items Excluded Based on Item-Total Correlation Coefficient and EFA Results**

**Point 4-1**: **4. Criterion Validity Interpretation.** The revised interpretation appropriately describes the effect sizes as "small to moderate" (Lines 549–552), which is a clear improvement over the original language. However, the Abstract still states "Criterion-related validity was supported by a significant positive correlation with physical activity (r = .19, p < .001)" (Lines 33–35) without any qualifier regarding effect size. A brief modifier (e.g., "a small but significant positive correlation") in the Abstract would improve consistency with the Discussion and prevent readers from overinterpreting the association based on the Abstract alone.

- **Response:** Thank you for your thoughtful comment. We revised the Abstract to describe the correlation with physical activity as a small but significant positive correlation and the correlation with sedentary time as a small-to-moderate negative correlation, thereby improving consistency with the Discussion.
- **Line 33-35:** Criterion-related validity was supported by a small but significant positive correlation with physical activity (r = .19, p < .001) and a small to moderate negative correlation with sedentary time (r = −.33, p < .001).

**Point 4-2**: **4. Criterion Validity Interpretation.** Additionally, the authors argue that the small effect sizes are "theoretically meaningful, given that the SBRS is designed to assess regulatory capacity rather than direct behavioral frequency" (Lines 551–552). While this argument has some merit, it would be strengthened by citing comparable effect sizes from validation studies of other self-regulation or self-efficacy scales. Without such comparative context, the justification remains somewhat circular.

- **Response:** We revised the Discussion by adding relevant validation studies of self-regulation and self-efficacy scales and clarified that similarly modest associations with behavioral outcomes have been reported in prior research.
- **Line 566-570:** The SBRS demonstrated a statistically significant positive correlation with physical activity and a negative correlation with sedentary time. Although the observed effect sizes were small to moderate, similar magnitudes have been reported in validation studies of self-regulation and self-efficacy measures related to sedentary behavior and physical activity, supporting the theoretical meaningfulness of the present findings [39, 40].

**Point 5: 5. Cultural Validity**. The expanded discussion (Lines 527–539) provides a general argument that the SBRS items capture floor-sitting-related regulatory behaviors indirectly. However, the argument remains speculative because: (a) No empirical evidence is presented to demonstrate that respondents' SBRS scores differ as a function of floor-sitting frequency or that items function differently across sedentary contexts (e.g., chair-based vs. floor-based). (b) The claim that items were "intentionally designed to capture regulatory behaviors that are prevalent in sedentary contexts characteristic of Korean daily life" (Lines 528–529) would benefit from at least one or two concrete examples mapping specific items to floor-sitting-related regulation. I recognize that a full analysis of differential item functioning may be beyond the scope of this study, but the authors should temper their claims about cultural sensitivity by acknowledging that this remains an empirical question to be tested in future research, rather than presenting it as an established feature of the SBRS.

- **Response:** Thank you for your valuable comment. We revised the Discussion to present this point more cautiously, framing the relevance of the scale to floor-sitting-related contexts as a possible interpretation rather than a definitive empirical conclusion.
- **Line 538-555:** Although the final 12 items of the SBRS do not explicitly reference floor sitting, they may still be relevant to sedentary contexts characteristic of Korean daily life. In Korea, sedentary behavior often occurs not only in chair-based settings but also in floor-seated postures during activities such as dining, leisure, and social interaction. This contextual relevance may be particularly important in Korea, where objectively measured sitting time appears to be shaped by demographic and everyday contextual correlates, suggesting that sedentary behavior assessment may benefit from considering how sitting is embedded in daily routines rather than relying solely on generic duration-based indicators [17]. For example, items addressing frequent postural adjustment, stretching, standing up in response to discomfort, and intentional movement during prolonged sitting reflect self-regulatory strategies commonly used to mitigate the physical strain associated with floor-based and low-seated positions. From this perspective, the potential cultural relevance of the SBRS may lie in its focus on how individuals manage bodily discomfort and initiate movement within seated contexts, rather than in any direct assessment of floor-sitting behavior itself. Accordingly, the scale may have relevance within the Korean sociocultural context while retaining possible applicability to other cultures with similar sedentary practices. However, whether these items function similarly across chair-based and floor-based sedentary settings remains an empirical question that should be examined in future research.

**Point 6**: Abstract, Line 37: The phrase "the SBRS proved to be a reliable and valid instrument" is strong language for a scale with sub-threshold AVE. Consider revising to "the SBRS demonstrated preliminary evidence of reliability and validity" to maintain consistency with the developmental framing adopted throughout the revised Discussion.

- **Response:** In response, we revised the Abstract to use more cautious language. Specifically, the phrase “the SBRS proved to be a reliable and valid instrument” was changed to “the SBRS demonstrated preliminary evidence of reliability and validity” in order to maintain consistency with the interpretation adopted in the revised Discussion.
- **Line 37-39:** Overall, the SBRS demonstrated preliminary evidence of reliability and validity for assessing behavioral and environmental strategies to reduce sedentary time.

**Point 7**: Lines 116–118: The phrase "sedentary behavior regulation is conceptualized as an autonomously motivated process involving self-awareness of sedentary patterns, active behavioral practices to interrupt or reduce sedentary time, and preventive environmental structuring" still lists three components including self-awareness, which contradicts the statement two sentences later (Lines 123–126) that self-awareness is not an independent regulatory dimension. This internal inconsistency within the Introduction should be reconciled.

- **Response:** We agree that the phrasing created an internal inconsistency in the Introduction. Accordingly, we removed the contradictory sentence to ensure conceptual consistency in the revised manuscript.

**Point 8**: Table 4: The standard deviation for physical activity (PA) is reported as "3901.88 ± 59.14." An SD of 59.14 for a variable with a range of 0–46,350 MET-min/week appears very small. Please verify whether this is a typographical error.

- **Response:** Thank you for your careful review. We checked the reported value and confirmed that the standard deviation for physical activity was incorrectly entered due to a typographical error. The correct standard deviation is 5923.14 rather than 59.14. This error has been corrected in the revised table.

**Point 9**: Line 609: The Conclusions state "developed and preliminarily validated," which is appropriate and consistent with the revised framing. However, Line 617 states "providing a valid measurement tool," which reverts to a more definitive claim. This should be softened to maintain consistency.

- **Response:** Thank you for your helpful comment. We revised this sentence to use more cautious language and to reflect that the present study provides preliminary evidence supporting the SBRS as a measurement tool.
- **Line 641-643:** By conceptualizing sedentary behavior as modifiable and providing preliminary evidence for the SBRS as a measurement tool, this study offers academic and practical value.

**We sincerely appreciate the Editor and the reviewers for their careful reading of our manuscript and for their thoughtful and constructive comments. Their detailed suggestions have substantially strengthened the quality and clarity of this work. We are truly grateful for the time and scholarly effort they invested in helping us improve the manuscript.**
